# Gamma radiolytic stability of the novel modified diglycolamide 2,2′-oxybis(*N*,*N*-didecylpropanamide) (mTDDGA) for grouped actinide extraction

**DOI:** 10.1039/d1ra08761d

**Published:** 2022-04-25

**Authors:** Bart Verlinden, Karen Van Hecke, Andreas Wilden, Michelle Hupert, Beatrix Santiago-Schübel, Richard J. M. Egberink, Willem Verboom, Piotr M. Kowalski, Giuseppe Modolo, Marc Verwerft, Koen Binnemans, Thomas Cardinaels

**Affiliations:** Belgian Nuclear Research Centre (SCK CEN), Institute for Nuclear Materials Science Boeretang 200 2400 Mol Belgium karen.van.hecke@sckcen.be; Department of Chemistry, KU Leuven Celestijnenlaan 200F, P.O. Box 2404 3001 Leuven Belgium; JARA Energy, Center for Simulation and Data Science (CSD) Jülich Germany; Forschungszentrum Jülich GmbH, Institut für Energie- und Klimaforschung – Nukleare Entsorgung und Reaktorsicherheit (IEK-6) 52428 Jülich Germany; Forschungszentrum Jülich GmbH, Zentralinstitut für Engineering, Elektronik und Analytik (ZEA-3) 52428 Jülich Germany; Department of Molecules & Materials, Mesa+ Institute for Nanotechnology, University of Twente P.O. Box 217 7500 AE Enschede The Netherlands; Forschungszentrum Jülich GmbH, Institute of Energy and Climate Research: Theory and Computation of Energy Materials (IEK-13) 52428 Jülich Germany

## Abstract

Reprocessing of spent nuclear fuel aims at improving resource efficiency and reducing its radiotoxicity and heat production in the long term. The necessary separation of certain metal ions from the spent fuel solutions can be achieved using different solvent extraction processes. For the scenario of the EURO-GANEX process, the use of the new, modified diglycolamide 2,2′-oxybis(*N*,*N*-didecylpropanamide) (mTDDGA) was recently proposed to simplify the current solvent composition and reduce extraction of fission products. Before further developing the process based on this new ligand, its stability under ionizing radiation conditions needs to be studied. For this reason, gamma irradiation experiments were conducted followed by analyses with high performance liquid chromatography coupled to a mass spectrometer (HPLC-MS). The determined degradation rate of mTDDGA was found to be lower than that of the reference molecule *N*,*N*,*N*′,*N*′-tetra-*n*-octyl-diglycolamide (TODGA). Many identified degradation compounds of both molecules are analogues showing the same bond breaking, although also unreported de-methylation, double/triple de-alkylation and *n*-dodecane addition products were observed.

## Abstract 2

The radiolysis behavior of a new diglycolamide for solvent extraction of actinides and lanthanides was studied. The observed degradation rate was lower than for the reference molecule and 22 degradation compounds were identified.

## Graphical abstract

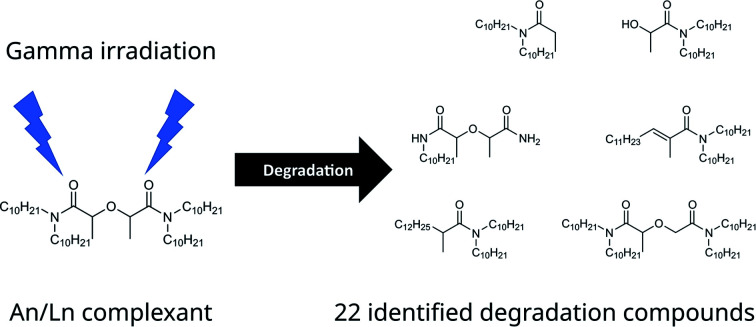

## Introduction

To meet humanities' increasing energy requirement, different sources of energy are employed. One of these sources of energy for electric power production is based on nuclear fission of ^235^U and ^239^Pu nuclei, generally known as nuclear power. Although being a low carbon dioxide emitting technology, the waste produced by this technology remains highly radiotoxic and produces heat for hundreds of thousands of years, making deep geological disposal essential for safe storage of the waste. Minor actinides and plutonium are the main contributors of radiotoxicity and heat production in the long term, defining requirements for storage facilities and repositories.^2,3^ Therefore, new concepts are investigated aiming at the use of plutonium and minor actinides as fuel in fast neutron spectrum reactors (*e.g.* generation IV reactors) and reducing the actinide inventory.^3–7^

The Grouped Actinide Extraction (GANEX) process was developed by the French Alternative Energies and Atomic Energy Commission (CEA) for the reprocessing of generation IV spent nuclear fuels and the homogenous recycling of actinides.^8^ It aims at extracting all actinides together and therefore avoids a separate Pu stream, which makes it a more proliferation resistant separation strategy.^9^ Because of the high initial abundance of uranium in dissolved spent fuel, the bulk amount of uranium is removed in a first separation cycle.^10,11^ This initial separation can be achieved by using *N*,*N*-di(ethyl-2-hexyl)isobutyramide (DEHiBA) in an industrial aliphatic diluent. The hydrometallurgical process for the U extraction from used nuclear fuel dissolved in nitric acid solutions was demonstrated on the laboratory scale; from genuine highly active liquid waste more than 99.99% of the initial U was recovered.^10^

The subsequent second CEA GANEX extraction cycle was also demonstrated.^11^*N*,*N*-Dimethyl-*N*,*N*-dioctyl-2-(2-hexyloxyethyl)-malonamide (DMDOHEMA) and di-(2-ethylhexyl)phosphoric acid (HDEHP) were used to co-extract the actinides. However, HDEHP contains phosphorus, which is undesirable as it complicates the disposal of the secondary cycle waste. In the collaborative European research projects ACSEPT, SACSESS and GENIORS, different research pathways for the second separation cycle have been followed.^12–15^ The CHALMEX process, developed by Chalmers University, uses a route based on a direct actinide co-extraction by 6,6′-bis(5,5,8,8-tetramethyl-5,6,7,8-tetrahydrobenzol-[1,2,4]-triazin-3-yl)[2,2]bipyridine (CyMe_4_–BTBP) and tri-*n*-butyl phosphate (TBP) in phenyl trifluoromethyl sulfone (FS-13), for which elaborate solvent composition optimization has been conducted.^15–21^

The current formulation of the EURO-GANEX process makes use of two extractants in odorless kerosene to first extract both the actinides and the lanthanides into the organic phase.^22–24^ For this first co-extraction, a solvent comprising 0.5 mol L^−1^ DMDOHEMA and 0.2 mol L^−1^*N*,*N*,*N*′,*N*′-tetra-*n*-octyl-diglycolamide (TODGA) was used.^22^ TODGA exhibits very high distribution ratios for actinides and lanthanides, but the addition of DMDOHEMA is essential to avoid third phase formation caused by the high Pu concentration.^25^ After the actinides and the lanthanides are extracted into the organic phase, a separation between both can be obtained by an actinide selective stripping step with an aqueous solution of 2,6-bis(5,6-di(sulfophenyl)-1,2,4-triazin-3-yl)pyridine (SO_3_–Ph–BTP) and acetohydroxamic acid (AHA).^23,26^

Although, the EURO-GANEX process reached a technology readiness level (TRL) of 4–5 in its evaluation during the SACSESS project, there are still fundamental improvements possible.^7,27^ In its current formulation, the combination of TODGA and DMDOHEMA extracts fission products such as Zr, Pd, Mo, and Tc.^28^ Extraction of Zr and Pd can be reduced by using *trans*-cyclohexane-1,2-dinitrilotetraacetic acid (CDTA) as a masking agent.^28,29^ The use of a mixed solvent with two extractants poses difficulties during solvent regeneration. Therefore, an organic solvent containing only one extractant would be easier to manage and is therefore preferable. Ideally, a single extractant, capable of extracting high metal (Pu) concentrations, would be used.^30^ When this organic phase degrades under the strong ionizing conditions, a less complex initial mixture would result in a less diverse mix of degradation compounds (DCs), facilitating regeneration.

**Fig. 1:**
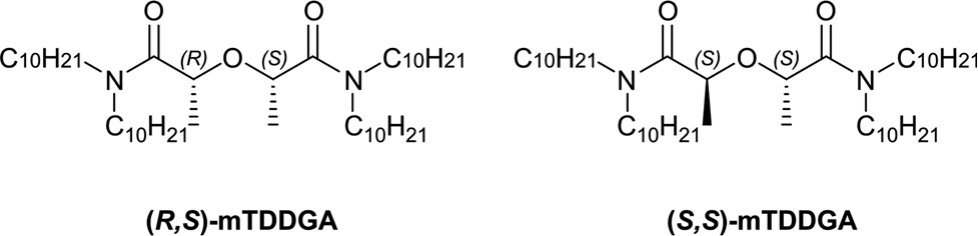
Chemical structures of (*R*,*S*) and (*S*,*S*)-mTDDGA.

To simplify the current version of the EURO-GANEX solvent extraction system, the new modified tetradecyl diglycolamide 2,2′-oxybis(*N*,*N*-didecylpropanamide) (mTDDGA) was developed and was shown to be a promising candidate for application in the process.^30^ The molecular structure of mTDDGA is shown in Fig. 1. Also, simplification of the system requires only the synthesis (and its costs) of one compound and will decrease the number of possible degradation products during operation. To increase the loading capacity (mainly for plutonium) of the solvent without third phase formation or precipitation, longer alkyl chains are used (decyl instead of octyl compared to TODGA) and a higher concentration of the extractant is used.^31^ The high concentration of a diglycolamide (to further increase the loading capacity) would result in very high distribution ratios for the extraction of actinides and lanthanides. This could possibly lead to issues with stripping and extraction of significant amounts of fission and corrosion products.^31^ For this reason, the diglycolamide backbone was methylated, reducing its complexing strength.^32–34^ As can be seen from Fig. 1, there are two different possible orientations of the methyl groups. Previously it was shown for Me_2_-TODGA that distribution ratios for trivalent actinides and lanthanides differed up to two orders of magnitude between the two different diastereomers.^34^ The Me_2_-TODGA extractant with both methyl groups orientated in the same direction was shown to yield better extraction capabilities compared to the extractant with orientation of the methyl groups in opposite directions. This difference was attributed to differences in the complexation of nitrate ions.^34^ Also for mTDDGA, these diastereomers exist, and similar differences in extraction behavior can be expected.

To evolve towards further application of mTDDGA, it is essential to evaluate the molecule's stability under ionizing radiation conditions. This is a necessary step because the organic phase will be in close contact with highly radioactive solutions and the extractant will form complexes with actinides and lanthanides. A ligand which quickly degrades and/or produces radiolysis products which tend to drastically change the extraction behavior, is not desirable because the organic phase would need excessive regeneration, increasing costs and the amount of waste.^35^

Radiolytic stability studies on different diglycolamides were conducted using gamma irradiation.^1,36–42^ This allows for easy sampling and analysis because the exposed solutions themselves are not radioactive after removal from the irradiation field. In the past, the focus was mainly on TODGA, for which dose constants were determined under varying conditions and many degradation compounds were identified by Sugo *et al.*^37^ Further research on TODGA was conducted by Galàn *et al.* using high performance liquid chromatography coupled to a mass spectrometer (HPLC-MS) for even more sensitive identification of decomposition products and for quantification.^43^ Methylated derivates of TODGA (Me-TODGA and Me_2_-TODGA) were found not to be consistently more radiation resistant.^36^ On the contrary, it was found that Me-TODGA shows faster degradation under gamma irradiation.^36^ This finding disproved the hypothesis that methylation of the ether backbone could protect the DGA sterically for radical attacks.^36,44^ When studying the short alkyl chained hydrophilic DGAs, Wilden *et al.* denoted a positive correlation between molecular weight of the DGA and its radiolytic stability.^45^ Considering this idea, mTDDGA has the potential to have an improved radiolytic stability compared to TODGA. Alpha radiation induced ligand degradation is generally studied to a lesser extent, as it poses additional challenges towards the determination of the absorbed dose by the solvent and especially the analysis of the irradiated samples.^46–49^ This is because samples exposed to strong alpha radionuclides remain highly radioactive. Previously, it was shown that high linear energy transfer (LET) irradiation with alpha radiation leads to a slower degradation of TODGA, probably because recombination of the formed radical species occurs more often.^46^ The same effect was also observed for octyl(phenyl)-*N*,*N*-diisobutylcarbamoylmethyl phosphine oxide (CMPO), another amide type actinide/lanthanide extractant.^49^

Dissolution of used nuclear fuel and separation of the actinides with solvent extraction systems involves the presence of high concentrations of nitric acid. For this reason, the presence of aqueous phases containing nitric acid is an important factor when studying radiolysis of DGAs. Radiolysis of water causes the presence of radical species such as ˙OH, H˙ and radiolysis of nitric acid leads to nitrite formation.^50^ Nitric acid is extracted into the organic phase by DGAs and is therefore potentially more relevant.^51^ Hydrolysis of TODGA by nitric acid, was shown to be not significant, as well as the influence of nitric acid on gamma irradiation in multiple studies, also when comparing DGAs with different alkyl sidechains.^37,39,41^ Galán *et al.*, however, observed a protective effect of nitric acid on TODGA during longer irradiations up to 1000 kGy.^43^ Also Kimberlin *et al.* reported a slight protective effect of nitric acid.^52^ For CMPO, nitric acid showed a strong protective effect.^49^

Also computational studies on DGA radiolysis have been conducted to support experimental findings. Koubský *et al.* published density functional theory (DFT) studies in which reaction profiles and bond orders of TODGA and its methylated derivatives were evaluated.^53,54^ The central ether bonds were determined to be the weakest bonds. The analysis by the Fukui functions shows that the hydrogen atoms on the carbon atoms next to the ether bond are prone to a radical attack, which correlates to the previous general observations of products resulting from the ether bond breaking.^55^ In a recent study by Verlinden *et al.*, a destabilization of both ether bonds was observed for single methylated TEDGA.^56^ Thermodynamics of the solvent proved to be decisive for the initially suggested hydrogen abstraction degradation pathway. The study reports that calculations of radical Fukui functions, indicators for radical attack, can be performed using standard generalized gradient approximation based DFT functionals.^55,56^

In this paper, we present a study on the radiolytic degradation of mTDDGA. First, mTDDGA radiolysis is compared quantitatively to the radiolysis of TODGA, which serves as the reference molecule. Degradation compounds of mTDDGA were identified and compared to similar previous studies on other diglycolamides using high resolution HPLC-MS. Additionally, analysis by the Fukui functions indicates the similarity of reactivity between Me_2_-TODGA and mTDDGA towards radical attack.

## Experimental

### Chemicals

The extractant mTDDGA was provided by the University of Twente. The purity was confirmed by ^1^H-NMR (>97%), as well as the ratio of both diastereomers of mTDDGA (3.5 : 1, (*R*,*S*)-mTDDGA : (*S*,*S*)-mTDDGA(2)). Solutions were prepared by weighing the required amount of mTDDGA followed by dilution with *n*-dodecane (GPR Rectapur purchased from VWR). Nitric acid solutions were prepared by dilution of 65% nitric acid (p.a.) purchased from Merck.

### Gamma irradiation

Gamma irradiations up to 1297 kGy with ^60^Co sources were conducted at the BRIGITTE irradiation facility of SCK CEN. The dosimetry and methodology are described elsewhere.^1^ The average dose rate in the irradiation chamber was determined as 9.44 kGy h^−1^ at the samples' positions using Red Perspex dosimeters from Harwell. Absorbed doses for the samples were determined by dosimetry at the exact irradiation positions. After irradiation, samples were centrifuged for 5 min at 3250 rpm to ensure that samples of the organic phase did not contain precipitates which were present in samples irradiated in contact with nitric acid.

### Quantification

Analysis of the irradiated ligand solutions was performed using High Performance Liquid Chromatography coupled with Mass Spectrometry (HPLC-MS).^38,39,41,45,57^ For quantification purposes in complex (irradiated) mixtures, an HPLC-MS/MS method with ESI in positive modus was developed using a Qtrap6500 instrument (ABSciex, Darmstadt, Germany) coupled with an Agilent 1260 HPLC (Agilent, Waldbronn, Germany).

Chromatographic separation was performed on an Accucore-150-C4 (4.6 × 100 mm; 2.6 µm particle size; Thermo Scientific, Waltham, Massachusetts, USA). Column temperature was kept at 50 °C. The mobile phases consisted of 85/15 methanol/H_2_O and 0.1% formic acid (solvent A) and acetonitrile + 0.1% formic acid (solvent B) a flow-rate of 700 µL min^−1^. Sample injection volume was 10 µL. At the beginning of the run was an isocratic step of 5% B (0 to 10 min) followed by an increase to 95% B within 8 min, which was held for 5 min for cleaning with 95% B. The gradient returned to 10% B within 0.1 min and equilibrated the system for 5 minutes.

For the detection, the Multiple Reaction Monitoring (MRM) method was used with optimized method parameters. Mass spectrometer settings were: curtain gas (N_2_) 40 arbitrary units (a.u.), temperature of the source 350 °C, nebulizer gas (N_2_) 80 a.u., heater gas (N_2_) 40 a.u., and ionspray voltage (IS) 4500 V. Quantitation after HPLC separation was performed using ESI-MS/MS detection in multiple reaction-monitoring (MRM) mode, with *m*/*z* = 721.3 as the parent molecule and *m*/*z* = 396.2 and *m*/*z* = 296.2 as the product ions. Data acquisition and processing were carried out using the software Analyst 1.6.1 (ABSciex, Darmstadt, Germany). For quantification the software Multiquant (ABSciex, Darmstadt, Germany) was used. The calibration curve showed linearity between 2 and 100 nM mTDDGA with a correlation coefficient of *R*^2^ = 0.9993.

The measured concentrations in irradiated samples were used to calculate the dose constant for a certain ligand, irradiated under specific conditions. The reason behind this is that the ligand degradation follows (pseudo) first order kinetics. This first order reaction rate can be used to express the concentration (*C*) of the ligand as a function of the absorbed dose *D* using the following equation:^58^

1 *C* = *C*_0_e^*dD*^

This allows for an easy approach to calculate the dose constant (*d*) as the slope of the linear fit of the natural logarithm of the ligand concentration as a function of the absorbed dose.

Expressing the degradation rate in terms of the more traditional *G* value is challenging in case of pseudo first order reaction rates, since this figure-of-merit depends on the initial concentration of the solute. However, for the exponential decrease of the concentration it is possible to define *G*_0_, the *G* value when the absorbed dose equals zero (*C* = *C*_0_).^59^

2 *G*_0_ (mol J^−1^) = *d* (Gy^−1^) × *C*_0_ (mol kg^−1^)

For consistency and to compare to previous studies, in this research we use dose constants to evaluate the mTDDGA stability under ionizing conditions.

### Identification of radiolysis products

Qualitative analyses for compound identification were performed using a hybrid linear ion trap FTICR (Fourier-Transform Ion Cyclotron Resonance) mass spectrometer LTQFT (Linear Tandem Quadrupole Fourier Transform) Ultra™ (Thermo Fisher Scientific, Bremen, Germany) coupled with an Agilent 1200 HPLC system (Agilent, Waldbronn, Germany). The chromatographic conditions were the same as in quantitative HPLS-MS/MS analysis. The mass spectrometer was first tuned and calibrated in the positive mode following the standard optimization procedure for all voltages and settings: source type: ESI, ion spray voltage: 3.8 kV, capillary voltage: 37.00 V, tube lens; 130.00 V, capillary temp.: 275.00 °C, sheath gas flow: 60.00. Mass spectra were recorded in full scan from 100 to 1000 Da with a resolution of 100 000 at *m*/*z* = 400. All data were processed using the XCalibur software version 2.0.

Semi-quantitative data analysis was performed using MZmine 2.^60^ Chromatograms for selected ions were reproduced from the high-resolution spectra as a function of elution time, with high selectivity (*m*/*z* = 0.01) and a threshold intensity of 10^3^ counts to remove noise. The peak areas of the clearly separated signals were used to perform the semi-quantitative analyses and follow the species' presence in irradiated samples. For all reported degradation products, the measured chemical formula deviated less than 0.5 ppm from the predicted one.^61^

### Computations

The Quantum ESPRESSO package was used for the DFT calculations with the PBE exchange–correlation functional, ultrasoft pseudopotentials and plane-wave cutoff energy of 30 Ryd in vacuum.^62–64^ The molecular structure was computed in large simulation boxes so the repeated images were separated by at least 10 Å. Chemdraw 14.0 was used for the creation of input coordinates for further geometrical optimization. Values for the radical Fukui function were derived from the resulting electron density of the DFT output of the molecules with an added and subtracted electron.^65^

## Results and discussion

### Determination of dose constants for mTDDGA

**Fig. 2:**
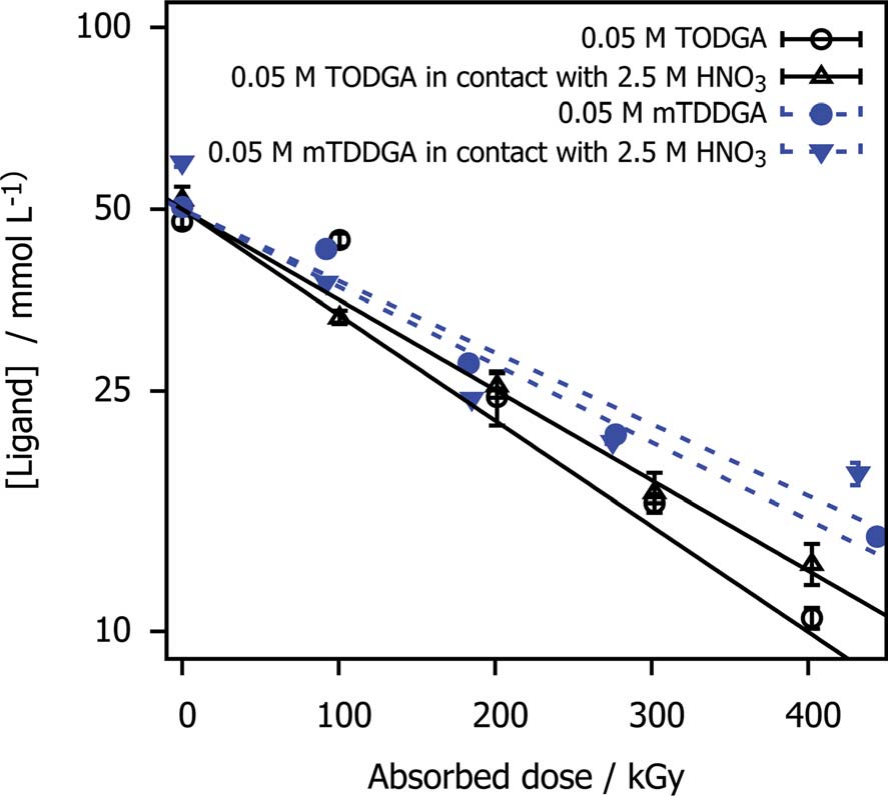
TODGA and mTDDGA concentrations as a function of the absorbed dose and presence of an HNO_3_ aqueous phase during irradiation. The data for TODGA was taken from Verlinden *et al.*^1^ The initial concentration was 0.05 mol L^−1^ for both ligands. Error bars represent the standard deviation of three analysis runs. The *Y*-axis is scaled with the natural logarithm.

The concentration of mTDDGA in samples irradiated with or without contact to an aqueous phase of 2.5 mol L^−1^ HNO_3_ was measured as a function of the absorbed dose and compared to the reference molecule TODGA. The results are shown in Fig. 2 and the derived dose constants are given in Table 1. The dose constant for 0.05 mol L^−1^ TODGA in *n*-dodecane, calculated from Verlinden *et al.*,^1^ is similar to those published by Zarzana *et al.*^39^ (−4.1 × 10^−3^ kGy^−1^) and Sugo *et al.*^66^ (−4.5 × 10^−3^ kGy^−1^).

**Table 1:** Determined dose constants, resulting from the linear least square regression of the natural logarithm of the measured concentrations as a function of the absorbed dose. Dose constants for TODGA were based on data from Verlinden *et al.*^1^ The intersection with the *y* axis for the linear fitting was set at the initial concentration, prepared by accurate weighing

Sample	*d* (×10^−3^ kGy^−1^)
0.05 mol L^−1^ TODGA	−3.7 ± 0.2
0.05 mol L^−1^ TODGA in contact with 2.5 mol L^−1^ HNO_3_	−3.4 ± 0.1
0.05 mol L^−1^ mTDDGA	−2.9 ± 0.1
0.05 mol L^−1^ mTDDGA in contact with 2.5 mol L^−1^ HNO_3_	−2.8 ± 0.3

Compared to TODGA, mTDDGA shows a slightly lower dose constant for the samples irradiated, indicating higher stability against radiolysis. This observation is in agreement with what could be expected from the results of previous work of Galàn *et al.*^36^ in which double methylated TODGA derivates showed a lower dose constant. They reported a lower dose constant (−3.0 ± 0.2) × 10^−3^ kGy^−1^ for the irradiation of the neat organic phase containing 0.05 mol L^−1^ Me_2_-TODGA derivative. However, they observed an increase in dose constant (to (−5.3 ± 0.4) × 10^−3^ kGy^−1^) for Me_2_-TODGA samples in contact with 2.5 mol L^−1^ HNO_3_ solution compared to the ones without nitric acid contact, which was not observed in this study.^36^ Our findings are consistent with previous observations that the DGAs' radiation resistance increases with the molecular weight.^40,45^

### Identification of degradation compounds

**Fig. 3:**
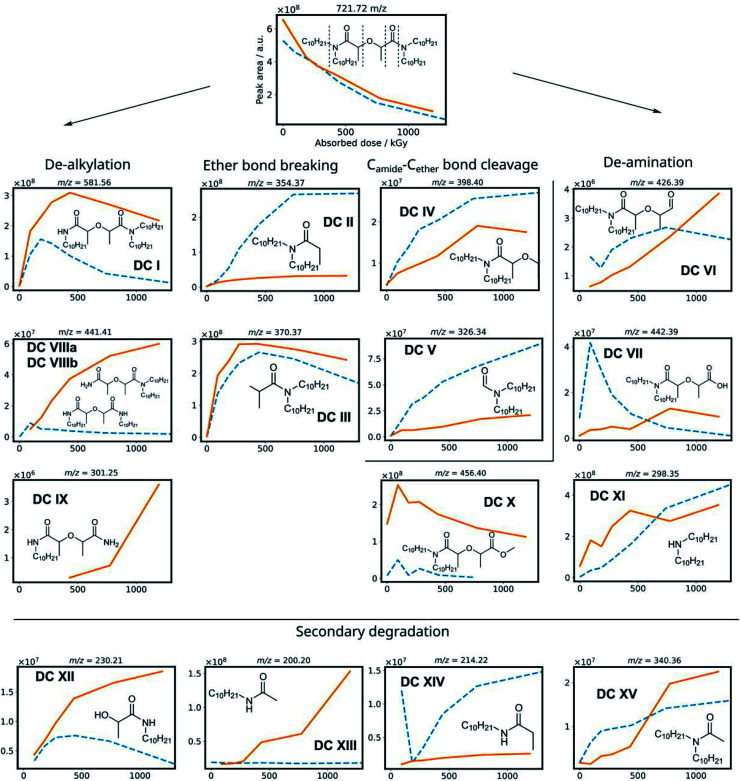
Scheme with degradation compounds of mTDDGA, with the peak area of their corresponding [M + H]^+^ species as a function of the absorbed dose for the irradiation in contact with 2.5 mol L^−1^ HNO_3_ (solid, orange) and the neat organic phase (dotted, blue). Chemical structures show the degradation compounds [M], while the *m*/*z* ratio corresponds to the protonated [M + H]^+^ species.

To obtain information from a mixture of degradation compounds and to accurately determine their chemical formula from the mass over charge ratio (*m*/*z*), high resolution MS is essential since multiple compounds can seemingly have the same *m*/*z* ratio on a unit level. This allows for predicting chemical formulas of detected ions.^67^ Semi-quantitative data was obtained from the chromatogram peak areas of the [M + H^+^] ions within a *m*/*z* of 0.01 window. The MS intensities of the different degradation products are shown in Fig. 3 as a function of the absorbed dose for the irradiation of mTDDGA solutions in contact with an aqueous nitric acid solution. Different molecules have different ionization potentials and therefore different ionization efficiencies, which makes the interpretation of these results only semi-quantitative. Suitable calibration standards were not available for the degradation compounds. Also, the intensity may vary depending on the total ion count at the moment of elution, as a consequence of *e.g.*, space charging effects.^68,69^ For most degradation products, also sodium and ammonium adducts could be observed, although their intensities were generally lower than the protonated ones.

**Fig. 4:**
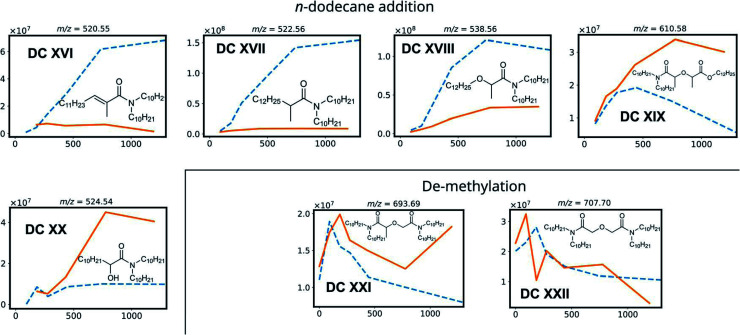
Degradation compounds of mTDDGA, with the peak area of their corresponding [M + H]^+^ species as a function of the absorbed dose for the irradiation in contact with 2.5 mol L^−1^ HNO_3_ (solid, orange) and the neat organic phase (dotted, blue). Chemical structures show the degradation compounds [M], while the *m*/*z* ratio corresponds to the protonated [M + H]^+^ species.

Based on previous studies on the radiolysis of diglycolamides, several main degradation compounds were expected to be produced during ^60^Co gamma irradiation. The results of the data analysis for these most frequently reported compounds are shown in Fig. 3 and 4. The peak areas of the protonated degradation compounds are shown as a function of the absorbed dose. This gives a clear indication whether the molecules were already present in the unirradiated samples or were formed during irradiation. The retention time of each peak is shown in Table 2.

**Table 2:** Retention time (RT) of the chromatographic peaks. If there are two separated peaks for both diastereomers, two retention times are shown

	*m*/*z* ([M + H]^+^)	RT 1 (min.)	RT 2 (min.)
DC I	581.56	7.82	8.34
DC II	354.37	4.07	
DC III	370.37	3.25	
DC IV	398.4	4.17	
DC V	326.34	3.30	
DC VI	426.39	3.51	3.89
DC VII	442.39	3.06	3.22
DC VIII	441.41	2.66	
DC IX	301.25	1.54	
DC X	456.4	3.66	3.84
DC XI	298.35	1.27	
DC XII	230.21	1.54	
DC XIII	200.20	1.69	
DC XIV	214.22	1.59	
DC XV	340.36	2.67	
DC XVI	520.55	11.13	
DC XVII	522.56	11.89	
DC XVIII	538.56	10.80	
DC XIX	610.58	10.51	
DC XX	524.54	11.72	
DC XXI	696.69	12.73	
DC XXII	707.70	13.34	
mTDDGA	721.72	13.87	

The mTDDGA molecules' [M + H]^+^ peak area decreases with increasing absorbed dose in an exponential manner, the same as observed in the quantitative results for the determination of the dose constants. The de-alkylation products DC I, DC VIIIa, and DC VIIIb are produced in the irradiated samples. The removal of one decyl group results in the single de-alkylation product (DC I). Diglycolamide molecules with two decyl groups removed, DC VIIIa and VIIIb, are shown to be formed as the absorbed dose further increases. A delayed formation for these compounds is observed compared to DC I, as DC VIIIa and DC VIIIb are radiolysis products of DC I. First, a significant amount of DC I needs to be produced to further degrade to the double de-alkylation products. The radiolysis of DC I is visible in Fig. 3, as the intensity decreases from 432 to 1192 kGy. DC I can also degrade *via* other degradation pathways (*e.g.* ether bond breaking), which can also lead to the formation of DC XII, DC XIII and DC XIV. For the product of triple de-alkylation (DC IX) a dose dependent peak area was observed starting at higher absorbed doses (>432 kGy) because it results from three consecutive de-alkylation reactions.

Degradation products of the C–O ether bond break (DC II and DC III) were found as main products of the irradiation of mTDDGA. In the literature, these products were found to be among the most important radiolysis products in irradiated diglycolamide solutions as well, together with other DCs resulting from degradation of the DGA backbone.^36,41,42,45,52^ A possible radiolysis mechanism based on hydrogen abstraction was suggested and studied computationally.^53,54,56^ Other DGA degradation mechanisms suggest solvent radical cations as the ones which transfer an electron or a hydrogen atom (H˙) to the DGA, which causes it to become unstable and further degrade.^70,71^

DC IV and DC V are products of the C_amide_–C_ether_ bond breaking. Their intensities increase with increasing absorbed dose. De-amination products (DC VI, DC VII, DC X and DC XI) show dose dependent peak areas, although destroying the conjugated amide system requires more energy as previously calculated for TEDGA.^56^ The suggested structure of DC X is the result of capping the carboxylic functional group with a ˙CH_3_ radical, which is formed during the irradiation of the *n*-dodecane diluent.^72,73^ The degradation compounds DC XII, DC XIII, DC XIV, and DC XV are the result of radiolysis of previously mentioned DCs, by breaking two bonds of mTDDGA consecutively. They can originate from a combination of de-alkylation and ether bond cleavage reactions. Additionally, molecules containing C_12_H_25_ additions were observed as DCs in the irradiated samples. It was previously shown that diluent radicals play an important role in DGA degradation, but here we also see corresponding radical addition products such as DC XVI, DC XVII, DC XVIII, DC XIX, and DC XX (Fig. 4). Their structure is like the other radiolysis products. It is possible that they reacted with dodecane radicals or recombined with a dodecane radical instead of ˙H or ˙OH.

The peak areas of identified DCs for samples irradiated in absence of any aqueous phase are also shown in Fig. 3 as a function of the absorbed dose. In general, similar DCs were found to be formed during irradiation, although there are some differences. Peak areas for de-alkylation, ether bond breaking, C_amide_–C_ether_ bond cleavage and de-amination products are showing analogous behavior. Intensities of the [DC IX + H]^+^ peak of the triple de-alkylation product were very low, below the noise threshold, and are therefore not visible in Fig. 3 in absence of an aqueous phase. For DC X, the methyl end-capped de-amination product, only an absorbed dose dependency was shown in the presence of an aqueous nitric acid solution. The ‘secondary degradation compounds’ DC XII, DC XV and XIV were found in neat organic samples too, while DC XIII could not be identified.

Interestingly, in the irradiated samples in absence of an aqueous nitric acid solution, also de-methylation products (DC XXI and DC XXII) were identified, as shown in Fig. 4. These were also present in the fresh solutions, but after absorbing a low dose of gamma irradiation (92 kGy) there is a strong increase in their [M + H]^+^ peak area. This indicates that de-methylation products are present in small amounts as byproducts of the synthesis, but more of these are also radiolytically produced. They could strongly interfere with the solvent extraction system as they probably have different complexing properties.

With regards to extraction properties, amides have the potency to enhance the extraction of tetravalent metal ions such as Pu and Zr in solvent extraction processes.^74^ All DCs which still have the diglycolamide backbone in their structure can be expected to be able to extract lanthanides and actinides, since the oxygen atoms' free electron pairs are responsible for coordination of the ligand molecules around the metal ions during solvent extraction.^75^ In particular, de-methylation products DC XXI and DC XXII are expected to extract trivalent metal ions very well, since methylation of DGAs leads to a lower complexation strength as reported for TODGA and TEDGA.^32,33,76^

### Chromatograms of irradiated mTDDGA solutions

**Fig. 5:**
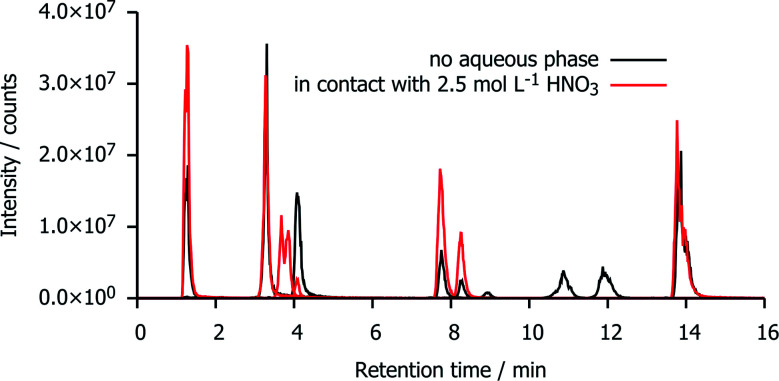
Combined ion chromatograms of the most intense signals in irradiated mTDDGA samples with an initial concentration of 0.05 mol L^−1^ mTDDGA irradiated with or without contact to an aqueous phase of 2.5 mol L^−1^ HNO_3_.

Fig. 5 shows a reconstruction of the ion chromatograms of the most intense signals in mTDDGA samples with an initial concentration of 0.05 mol L^−1^ mTDDGA irradiated to 400 kGy with or without contact to an aqueous phase of 2.5 mol L^−1^ HNO_3_. A similar approach was reported previously by Zarzana *et al.*^39^ This makes a direct comparison of the influence of the presence of an aqueous nitric acid solution during irradiation possible.

The signals corresponding to the single de-alkylation product (DC I) and the amine (DC XI) appear enhanced by the presence of an aqueous nitric acid solution. The reason that there are two chromatographic peaks for the de-alkylation product ion, is that there are two diastereomers present because the initial mTDDGA also consists of a mix of two diastereomers. For the same reason, also the ion of DC X appears as a double signal.

The abundance of the ion of DC II (C–O_ether_ bond breaking product) in the chromatogram is higher in the non-acidified sample. More remarkable is the presence of the ions with a retention time of 10–12 minutes (DC XVIII and DC XVII) in the neat organic phase. These are products of the C–O_ether_ bond break, capped with dodecyl radicals from the diluent, *n*-dodecane. Possibly, instead of being involved in addition reactions with *n*-dodecane, their radical intermediates or *n*-dodecane radicals are more likely to recombine with ˙OH radicals of the aqueous phase. The peak areas of these [M + H]^+^ ions increase as a function of the absorbed dose in both sample series, but their peak areas are one or two order of magnitudes larger in absence of an aqueous nitric acid solution.

### Calculation of the radical Fukui function

**Fig. 6:**
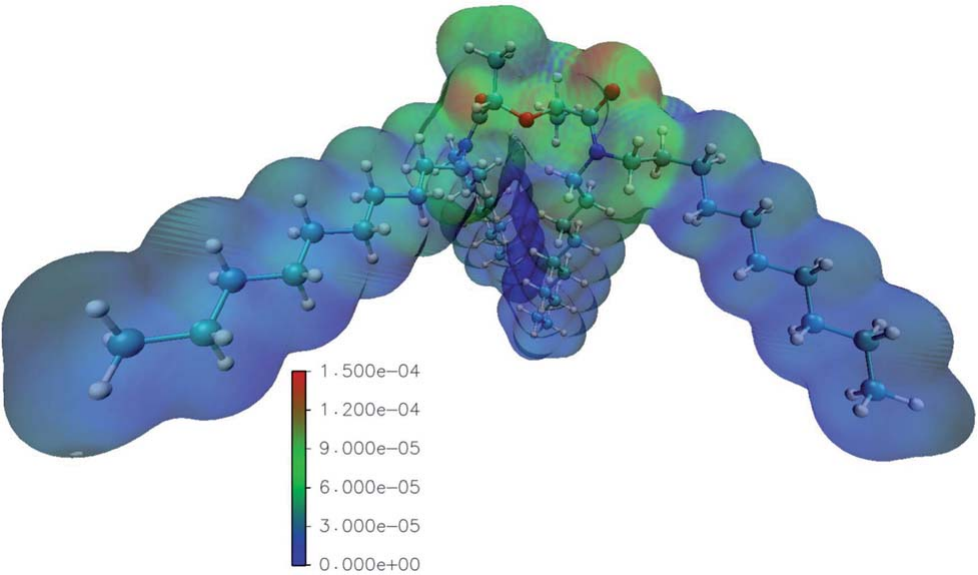
Visualization of the values of the radical Fukui functions on the isodensity surface of mTDDGA (in e a.u.^−3^), increasing from blue to green and red. Following colors are used for the representation of the atoms: white: hydrogen, red: oxygen, blue: nitrogen, cyan: carbon. The high isodensity (reddish colors) indicates parts of the molecule prone to the radical attack.

The reaction rate constants computed by Mezyk *et al.* imply the importance of reactions with the solvent radical cations in the case of TODGA.^70,71^ We thus computed the radical Fukui function of mTDDGA, as it is an excellent indicator for reactivity towards radicals.^53,55^ It provides highly valuable information, since during irradiation many radical species are produced in the considered organic phases. The computed radical Fukui function is shown in Fig. 6.^55^ Here we can identify the parts of the molecule that are most prone towards radical attacks, which is important information for DGA radiolysis.^53,54^ Previous experimental studies showed that organic radicals play a major role in these processes *via* electron or proton transfer mechanisms.^70,71^

The least reactive positions are found to be around the C–H bonds of the alkyl side chains. This agrees with the fact that we did not observe experimentally any products of reactions on the alkyl chains. For the C–H bonds of the DGA backbone, however, we clearly see higher values for the radical Fukui function, indicating susceptibility to the radical attack. Degradation *via* the hydrogen radical initiated hydrogen abstraction mechanism, as suggested by Koubský *et al.*, would indicate that hydrogen atoms next to the ether bond are more reactive, when degradation of the DGA is initiated by the hydrogen abstraction.^53^ Indeed, products of breaking the ether bond are abundantly observed, as discussed in the previous paragraphs. The highest values for the radical Fukui function are situated around the DGA backbone, where breaking bonds leads to most experimentally observed DCs.

The representation of the molecule with the isodensity surface surrounding the molecular structure in Fig. 6 illustrates the steric effect that the methyl groups on the backbone could provide towards radical attacks on the ether group. Hence, the lower degradation rate we observed for mTDDGA than for TODGA, is similar to the reported lower degradation rates for Me_2_-TEDGA compared to TEDGA and Me_2_-TODGA compared to TODGA.^36,45^ These computational results are in agreement with previous work on TODGA (and methylated derivates) by Koubský *et al.* and other own studies.^53,54,56^ Future calculations could include an evaluation of the reactivity in different environments. Especially, the presence of nitric, the formation of aggregates, adducts and different conformers of the diglycolamide are of particular interest.^52^

## Conclusions

The new diglycolamide mTDDGA shows an increased radiolytic stability towards gamma irradiation compared to TODGA, with no significant effect of the presence of nitric acid on the degradation rate. This is a confirmation that mTDDGA can be further used for the development of reprocessing processes. The effect of irradiation on extraction performance of solutions containing higher concentrations of ligand and nitric acid needs further investigation. The most important DCs, typical for DGA degradation, were identified and confirmed as radiolysis products with HPLC-MS. Based on these findings, we can conclude that mTDDGA undergoes similar radiolysis mechanisms as the other diglycolamides studied so far. However, in this study we have shown a dose dependence for the presence of unreported degradation products of which two originate from the formation of adducts with *n*-dodecane. Double and triple de-alkylation, as well as de-methylation products were determined as radiolysis products. These de-methylation products are potentially good extractants. Future research should also evaluate solvent extraction behavior of irradiated solvent extracted systems. The theoretical study supports the experimental findings, confirming the reactivity of the hydrogen atoms of the carbon atoms next to the ether bond and the steric effect of the methylation. This research opens perspectives to synthesize degradation products and use them for quantification and for evaluation of their extraction behavior towards actinides, lanthanides, and fission products.

## Author contributions

Bart Verlinden: conceptualization, investigation, formal analysis, visualization, writing – original draft. Karen Van Hecke: supervision, writing – review & editing. Andreas Wilden: conceptualization, writing – review & editing. Michelle Hupert: methodology, investigation, writing – original draft. Beatrix Santiago-Schübel: methodology, writing – review & editing. Richard J. M. Egberink: resources. Willem Verboom: resources, writing – review & editing. Piotr M. Kowalski: funding acquisition, software, writing – review & editing. Giuseppe Modolo: conceptualization, writing – review & editing. Marc Verwerft: supervision, writing – review & editing. Koen Binnemans: conceptualization, supervision, writing – review & editing. Thomas Cardinaels: conceptualization, funding acquisition, supervision, writing – review & editing.

## Conflicts of interest

There are no conflicts to declare.

## Supplementary Material

## References

[cit1] Verlinden B., Zsabka P., Van Hecke K., Verguts K., Mihailescu L.-C., Modolo G., Verwerft M., Binnemans K., Cardinaels T. (2021). Radiochim. Acta.

[cit2] Taylor R. , Reprocessing and Recycling of Spent Nuclear Fuel, Woodhead Publishing, Cambridge, 2015

[cit3] Salvatores M. (2005). Nucl. Eng. Des..

[cit4] Abram T., Ion S. (2008). Energy Policy.

[cit5] OECD-NEA , Strategies and Considerations for the Back End of the Fuel Cycle, Report 7469, Nuclear Energy Agency (NEA), Boulogne-Billancourt, France, 2021

[cit6] Alyapyshev M. Y., Babain V. A., Ustynyuk Y. A. (2016). Russ. Chem. Rev..

[cit7] Baron P., Cornet S. M., Collins E. D., DeAngelis G., Del Cul G., Fedorov Y., Glatz J. P., Ignatiev V., Inoue T., Khaperskaya A., Kim I. T., Kormilitsyn M., Koyama T., Law J. D., Lee H. S., Minato K., Morita Y., Uhlir J., Warin D., Taylor R. J. (2019). Prog. Nucl. Energy.

[cit8] Miguirditchian M. , Chareyre L., Sorel C., Bisel I., Baron P. and Masson M., presented in part at the ATALANTE 2008, Montpellier, 2008

[cit9] Goff K. M. , Fredrickson G. L. and Vaden D. E., in Advanced Separation Techniques for Nuclear Fuel Reprocessing and Radioactive Waste Treatment, ed. K. L. Nash and G. J. Lumetta, Woodhead Publishing, Oxford, 2011, ch. 5, pp. 120–137, 10.1533/9780857092274.1.120

[cit10] Miguirditchian M. , Sorel C., Camès B., Bisel I., Baron P., Espinoux D., Calor J.-N., Viallesoubranne C., Lorrain B. and Masson M., HA demonstration in the Atalante facility of the Ganex 1st cycle for the selective extraction of Uranium from HLW, Paris, 2009

[cit11] Miguirditchian M. , Roussel H., Chareyre L., Baron P., Espinoux D., Calor J.-N., Viallesoubranne C., Lorrain B. and Masson M., HA demonstration in the Atalante facility of the GANEX 2nd cycle for the grouped TRU extraction, Paris, 2009.

[cit12] Bourg S., Hill C., Caravaca C., Rhodes C., Ekberg C., Taylor R., Geist A., Modolo G., Cassayre L., Malmbeck R., Harrison M., de Angelis G., Espartero A., Bouvet S., Ouvrier N. (2011). Nucl. Eng. Des..

[cit13] Geist A., Taylor R., Ekberg C., Guilbaud P., Modolo G., Bourg S. (2016). Procedia Chem..

[cit14] Bourg S. , GENIORS Project Homepage, https://www.geniors.eu/, accessed 2021-04-27, 2021

[cit15] Halleröd J., Ekberg C., Löfström-Engdahl E., Aneheim E. (2015). Nukleonika.

[cit16] Aneheim E., Ekberg C., Fermvik A., Foreman M. R. S., Grűner B., Hájková Z., Kvičalová M. (2011). Solvent Extr. Ion Exch..

[cit17] Aneheim E., Ekberg C., Fermvik A., Foreman M. R. S., Retegan T., Skarnemark G. (2010). Solvent Extr. Ion Exch..

[cit18] Aneheim E., Ekberg C., Foreman M. R. S., Löfström-Engdahl E., Mabile N. (2012). Sep. Sci. Technol..

[cit19] Aneheim E., Ekberg C., Foreman M. R. S. (2013). Solvent Extr. Ion Exch..

[cit20] Lyseid Authen T., Wilden A., Halleröd J., Schneider D., Kreft F., Modolo G., Ekberg C. (2020). Solvent Extr. Ion Exch..

[cit21] Lyseid Authen T., Wilden A., Schneider D., Kreft F., Modolo G., Foreman M. R. S., Ekberg C. (2021). Solvent Extr. Ion Exch..

[cit22] Bell K., Carpentier C., Carrott M., Geist A., Gregson C., Hérès X., Magnusson D., Malmbeck R., McLachlan F., Modolo G., Müllich U., Sypula M., Taylor R., Wilden A. (2012). Procedia Chem..

[cit23] Carrott M., Bell K., Brown J., Geist A., Gregson C., Hères X., Maher C., Malmbeck R., Mason C., Modolo G., Müllich U., Sarsfield M., Wilden A., Taylor R. (2014). Solvent Extr. Ion Exch..

[cit24] Taylor R., Carrott M., Galán H., Geist A., Hères X., Maher C., Mason C., Malmbeck R., Miguirditchian M., Modolo G., Rhodes C., Sarsfield M., Wilden A. (2016). Procedia Chem..

[cit25] Brown J., McLachlan F., Sarsfield M., Taylor R., Modolo G., Wilden A. (2012). Solvent Extr. Ion Exch..

[cit26] Geist A., Müllich U., Magnusson D., Kaden P., Modolo G., Wilden A., Zevaco T. (2012). Solvent Extr. Ion Exch..

[cit27] Joly P. and Boo E., Roadmap: Actinide separation processes 2015, Euratom Research and Training Programme on Nuclear Energy within the Seventh Framework Programme, Paris, France, 2015

[cit28] Carrott M., Geist A., Hères X., Lange S., Malmbeck R., Miguirditchian M., Modolo G., Wilden A., Taylor R. (2015). Hydrometallurgy.

[cit29] Sypula M., Wilden A., Schreinemachers C., Malmbeck R., Geist A., Taylor R., Modolo G. (2012). Solvent Extr. Ion Exch..

[cit30] Malmbeck R., Magnusson D., Geist A. (2017). J. Radioanal. Nucl. Chem..

[cit31] Sasaki Y., Sugo Y., Suzuki S., Kimura T. (2005). Anal. Chim. Acta.

[cit32] Iqbal M., Huskens J., Verboom W., Sypula M., Modolo G. (2010). Supramol. Chem..

[cit33] Wilden A., Modolo G., Lange S., Sadowski F., Beele B. B., Skerencak-Frech A., Panak P. J., Iqbal M., Verboom W., Geist A., Bosbach D. (2014). Solvent Extr. Ion Exch..

[cit34] Wilden A., Kowalski P. M., Klass L., Kraus B., Kreft F., Modolo G., Li Y., Rothe J., Dardenne K., Geist A., Leoncini A., Huskens J., Verboom W. (2019). Chem.–Eur. J..

[cit35] Mincher B. J. , in Comprehensive Nuclear Materials, Elsevier Ltd, Amsterdam, 2012, ch. 5.15, vol. 5, pp. 367–388

[cit36] Galán H., Zarzana C. A., Wilden A., Nunez A., Schmidt H., Egberink R. J. M., Leoncini A., Cobos J., Verboom W., Modolo G., Groenewold G. S., Mincher B. J. (2015). Dalton Trans..

[cit37] Sugo Y., Sasaki Y., Tachimori S. (2002). Radiochim. Acta.

[cit38] Zsabka P., Van Hecke K., Wilden A., Modolo G., Hupert M., Jespers V., Voorspoels S., Verwerft M., Binnemans K., Cardinaels T. (2020). Solvent Extr. Ion Exch..

[cit39] Zarzana C. A., Groenewold G. S., Mincher B. J., Mezyk S. P., Wilden A., Schmidt H., Modolo G., Wishart J. F., Cook A. R. (2015). Solvent Extr. Ion Exch..

[cit40] Horne G. P., Wilden A., Mezyk S. P., Twight L., Hupert M., Stark A., Verboom W., Mincher B. J., Modolo G. (2019). Dalton Trans..

[cit41] Roscioli-Johnson K. M., Zarzana C. A., Groenewold G. S., Mincher B. J., Wilden A., Schmidt H., Modolo G., Santiago-Schübel B. (2016). Solvent Extr. Ion Exch..

[cit42] Hubscher-Bruder V., Mogilireddy V., Michel S., Leoncini A., Huskens J., Verboom W., Galán H., Núñez A., Cobos J., Modolo G., Wilden A., Schmidt H., Charbonnel M. C., Guilbaud P., Boubals N. (2017). New J. Chem..

[cit43] Galán H., Núñez A., Espartero A. G., Sedano R., Durana A., de Mendoza J. (2012). Procedia Chem..

[cit44] Tedder J. M. (1982). Angew. Chem., Int. Ed..

[cit45] Wilden A., Mincher B. J., Mezyk S. P., Twight L., Rosciolo-Johnson K. M., Zarzana C. A., Case M. E., Hupert M., Stark A., Modolo G. (2018). Solvent Extr. Ion Exch..

[cit46] Malmbeck R., Banik N. L. (2020). J. Radioanal. Nucl. Chem..

[cit47] Fermvik A. (2011). J. Radioanal. Nucl. Chem..

[cit48] Mincher B. J., Mezyk S. P., Elias G., Groenewold G. S., LaVerne J. A., Nilsson M., Pearson J., Schmitt N. C., Tillotson R. D., Olson L. G. (2014). Solvent Extr. Ion Exch..

[cit49] Mincher B. J., Mezyk S. P., Groenewold G. S. (2016). Procedia Chem..

[cit50] Nagaishi R. , Jiang P. Y., Domae Y. K. M. and Ishigure K., Radiolysis of Concentrated Nitric Acid Solutions, Tokyo, Japan, 1995

[cit51] Bell K., Geist A., McLachlan F., Modolo G., Taylor R., Wilden A. (2012). Procedia Chem..

[cit52] Kimberlin A., Guillaumont D., Arpigny S., Camès B., Guilbaud P., Saint-Louis G., Galán H., Berthon L. (2021). New J. Chem..

[cit53] Koubský T., Fojtíková J., Kalvoda L. (2017). Prog. Nucl. Energy.

[cit54] Koubský T., Lustinec J. (2018). J. Radioanal. Nucl. Chem..

[cit55] Fukui K. (1982). Angew. Chem., Int. Ed..

[cit56] Verlinden B., Van Hecke K., Wilden A., Modolo G., Binnemans K., Cardinaels T., Kowalski P. M. (2022). Radiat. Phys. Chem..

[cit57] Sharma J. N., Ruhela R., Singh K. K., Kumar M., Janardhanan C., Achutan P. V., Manohar S., Wattal P. K., Suri A. K. (2010). Radiochim. Acta.

[cit58] Mincher B. J., Curry R. D. (2000). Appl. Radiat. Isot..

[cit59] Mincher B. J., Arbon R. E., Knighton W. B., Meikrantz D. H. (1994). Appl. Radiat. Isot..

[cit60] Pluskal T., Castillo S., Villar-Briones A., Orešič M. (2010). BMC Bioinf..

[cit61] Pluskal T., Uehara T., Yanagida M. (2012). Anal. Chem..

[cit62] Giannozzi P., Baroni S., Bonini N., Calandra M., Car R., Cavazzoni C., Ceresoli D., Chiarotti G. L., Cococcioni M., Dabo I., Dal Corso A., de Gironcoli S., Fabris S., Fratesi G., Gebauer R., Gerstmann U., Gougoussis C., Kokalj A., Lazzeri M., Martin-Samos L., Marzari N., Mauri F., Mazzarello R., Paolini S., Pasquarello A., Paulatto L., Sbraccia C., Scandolo S., Sclauzero G., Seitsonen A. P., Smogunov A., Umari P., Wentzcovitch R. M. (2009). J. Phys.: Condens. Matter.

[cit63] Perdew J. P., Burke K., Ernzerhof M. (1996). Phys. Rev. Lett..

[cit64] Vanderbilt D. (1990). Phys. Rev. B: Condens. Matter.

[cit65] Parr R. G., Yang W. T. (1984). J. Am. Chem. Soc..

[cit66] Sugo Y., Izumi Y., Yoshida Y., Nishijima S., Sasaki Y., Kimura T., Sekine T., Kudo H. (2007). Radiat. Phys. Chem..

[cit67] Milman B. L. (2015). TrAC, Trends Anal. Chem..

[cit68] Ledford, Jr E. B., Rempel D. L., Gross M. L. (1984). Anal. Chem..

[cit69] Wong R. L., Amster I. J. (2007). Int. J. Mass Spectrom..

[cit70] Mezyk S. P., Mincher B. J., Dhiman S. B., Layne B., Wishart J. F. (2015). J. Radioanal. Nucl. Chem..

[cit71] Mezyk S. P., Horne G. P., Mincher B. J., Zalupski P. R., Cook A. R., Wishart J. F. (2016). Procedia Chem..

[cit72] Schulder R. H., Kuntz R. R. (1963). J. Phys. Chem..

[cit73] Földiák G. (1980). Radiat. Phys. Chem..

[cit74] Siddall T. H. (1960). J. Phys. Chem..

[cit75] Ansari S. A., Pathak P., Mohapatra P. K., Manchanda V. K. (2012). Chem. Rev..

[cit76] Klaß L., Wilden A., Kreft F., Wagner C., Geist A., Panak P. J., Herdzik-Koniecko I., Narbutt J., Modolo G. (2019). Solvent Extr. Ion Exch..

